# A Case of an Undifferentiated Squamous Cell Carcinoma Arising from an Epidermal Cyst

**DOI:** 10.1155/2013/469516

**Published:** 2013-02-10

**Authors:** Mai Tokunaga, Masami Toya, Yuichiro Endo, Akihiro Fujisawa, Miki Tanioka, Mayumi Kato, Yoshiki Miyachi

**Affiliations:** ^1^Department of Dermatology, Graduate School of Medicine, Kyoto University, 54 Shogoin-Kawara-cho, Sakyo, Kyoto 606-8507, Japan; ^2^Department of Dermatology, Takatsuki Red Cross Hospital, 1-1-1 Abuno, Takatsuki 569-1096, Japan

## Abstract

An epidermal cyst is a common benign subcutaneous tumor and rarely develops malignancy. We report a case of an undifferentiated cutaneous squamous cell carcinoma (SCC) that arose from an epidermal cyst on the left side of the neck. The epidermal cyst had rapidly increased in size and presented cauliflower-like tumor. Histological study revealed undifferentiated squamous cell carcinoma that was arising from the epidermal cyst.

## 1. Introduction

An epidermal cyst is a common benign subcutaneous tumor and rarely develops malignancy. We report a case of an undifferentiated cutaneous squamous cell carcinoma (SCC) that arose from an epidermal cyst on the left side of the neck.

## 2. Case Presentation

**Figure 1:**
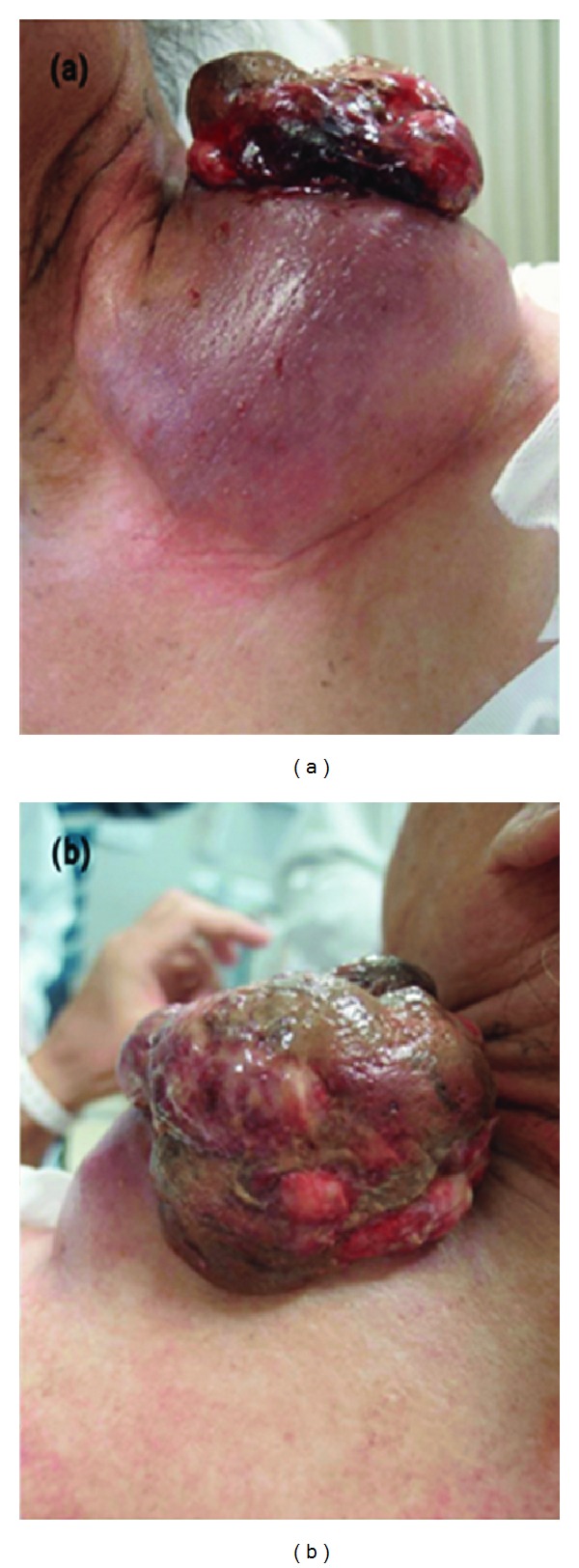
Most part of the tumor was confined into the epidermal cyst although cauliflower-like excrescence was observed from the fissure. (a) Anterior side. (b) Posterior side.

A 65-year-old man presented with a cauliflower-like tumor from a cyst on the left side of the neck (Figure 1). He reported that the cyst had existed for 35 years and had received occasional puncture. The cyst had gradually begun to increase in size and discharged bloody fluid 6 months prior to his hospital visit. The result of an excision biopsy from the tumor was not decisive because of the abundant hemorrhage and inflammation in the specimen. Magnetic resonance imaging and computed tomography (CT) revealed that although the tumor was 9 cm in diameter, it was completely encapsulated by the wall of the cyst and free of tumor invasion. Enhanced CT and ^18^F-FDG positron emission tomography (PET) showed neither distant metastasis nor lymph node metastasis. We suspected that the tumor was a SCC arising from an epidermal cyst.

**Figure 2:**
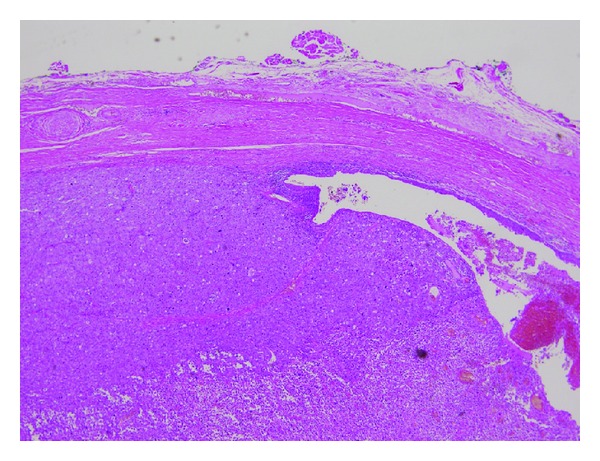
The squamous cell carcinoma developed within the epidermal cyst. (H and E ×40).

**Figure 3:**
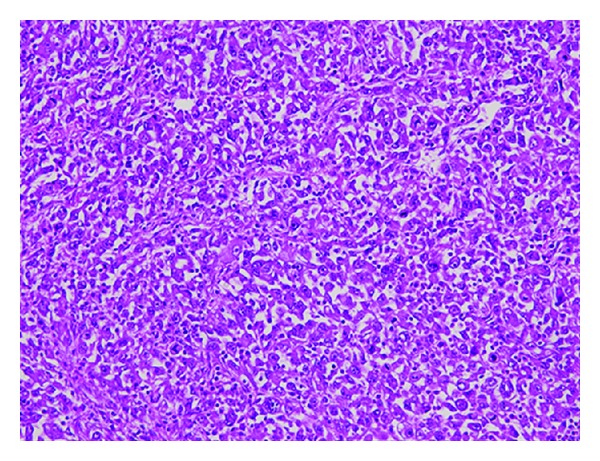
The tumor cells had eosinophilic cytoplasm and distinctive nucleoli that showed mitosis in some parts. (H and E ×400).

A surgical excision of the tumor with 1 cm margin was performed. The pathological examination was consistent with an undifferentiated SCC arising from an epidermal cyst (Figures 2 and 3). There was no sign of specific differentiation such as ductal formation or keratinization. Immunological staining showed that CAM5.2, vimentin, and EMA were positive while pankeratin, CK5, CK34*β*E12, S100, melan A, desmin, *α*SMA, and myogenin were negative. Therefore, we diagnosed a primary cutaneous SCC.

As the surgical margin was negative but close to the bottom of the cyst, postoperative radiation therapy is additionally planned.

## 3. Discussion

Reported rates of SCC arising from epidermal cysts range from 0.011 to 0.045% [1, 2]. There is a report that among the 19 case patients aged 21–80 (mean 43.2), 13 were men and 6 were women [2]. Most frequently affected region is the head and neck (42.1%) [2]. Lesion size ranged from 1.5 cm to 13 cm (mean 5.7 cm), whereas the duration of the lesions ranged from 2 to 480 months (mean 101 months) [2]. Metastasis was found in 3 cases which presented aggressive disease and death within 5 to 10 months [2]. 

The nature of the stimulus for malignant transformation in an epidermal cyst is uncertain [3]. It has been suggested that chronic irritation of the lesion could be a triggering factor [2]. Also there is growing evidence that human papillomavirus (HPV) infection may play a role in the development of nonmelanoma skin cancer [3, 4]. 

Primary treatment for a neoplastic cystic lesion is wide excision with adequate margin [3]. It is proposed that minimal margins of excision are 4 mm for all but high-risk tumors, for which at least a 6 mm margin is recommended [3]. 

In cutaneous SCC, tumor size larger than 2 cm, gender, preceding lesions, histological findings such as the degree of the differentiation, and location of tumors have been reported as prognostic factors of local recurrence, metastasis, and disease-specific death [1, 3, 5, 6]. The poor prognostic factors of SCC in this case were male, the poor differentiation, the tumor size, and rapid growth, which meant that this case had a high risk of metastasis and disease-specific death. Considering the result that the SCC microscopically invaded into cyst wall, we added postoperative radiation therapy. We need to watch for local recurrence and metastasis carefully.
